# Innovative advances in microalgal high-value products: unlocking potential from bioprocessing to bioindustrial applications

**DOI:** 10.3389/fbioe.2026.1737889

**Published:** 2026-03-26

**Authors:** Abdulrahman Alqahtani, Bayan Altoaimi

**Affiliations:** Saudi Food and Drug Authority (SFDA), Riyadh, Saudi Arabia

**Keywords:** carotenoids, high-value products, industrial biotechnology, microalgae, polyunsaturated fatty acids, regulatory readiness, systems-level bioprocessing

## Abstract

Microalgae are increasingly recognized as industrial biotechnology platforms for the sustainable production of high-value products (HVPs), including carotenoids, polyunsaturated fatty acids (PUFAs), polysaccharides, and phycobiliproteins. Their transition from exploratory biomass resources to precision metabolite-focused biofactories reflects growing industrial and regulatory consolidation. This review adopts a product-oriented perspective that moves beyond biomass productivity to evaluate how biological regulation, cultivation strategies, downstream processing, safety assessment, and regulatory readiness collectively determine industrial feasibility and commercialization potential. Rather than treating these components independently, the analysis frames them as interconnected determinants within a systems-level bioindustrial design framework. Emphasis is placed on stress-responsive biosynthesis, extraction and purification bottlenecks, mixotrophic cultivation, and strain engineering approaches as key enablers for improving productivity, process robustness, and cost performance. Carotenoids and omega-3 PUFAs are identified as the most industrially mature microalgal HVPs, supported by scalable production systems and regulatory acceptance, whereas polysaccharides and phycobiliproteins are highlighted as emerging products with expanding bioindustrial relevance. This comparative positioning underscores that industrial maturity depends not solely on biological potential, but on coordinated optimization across strain design, cultivation stability, downstream compatibility, and compliance pathways. By integrating technological innovation with safety and regulatory considerations, this review provides a coherent framework to support scalable, compliant, and sustainable microalgal biomanufacturing. Overall, the synthesis advances a rational roadmap for translating microalgal HVPs from laboratory optimization to economically viable and regulation-ready industrial deployment.

## 1 Introduction

Microalgae have emerged as a versatile biological resource for the generation of high-value products (HVPs), notably carotenoids, polyunsaturated fatty acids (PUFAs), polysaccharides, and phycobiliproteins, with growing relevance across industrial biotechnology sectors. Their metabolic diversity, capacity for controlled cultivation, and compatibility with engineered bioprocess systems position them as programmable biofactories rather than mere biomass sources. Their rapid growth rates, ability to utilize saline water or wastewater streams, and minimal land requirements position them as sustainable alternatives to conventional agricultural and petrochemical production systems, particularly in water-limited regions (Yu et al., 2024). These attributes align with circular bioeconomy principles and facilitate integration into resource-efficient production frameworks. Microalgal HVPs including carotenoids, PUFAs, polysaccharides and phycobiliproteins exhibit a convergence of biological functionality and industrial versatility, enabling applications in foods, cosmetics, and pharmaceuticals while offering advantages in sustainability, safety, and functional performance. Importantly, their industrial value depends not solely on metabolite presence but also on coordinated alignment of biosynthetic regulation, cultivation control, downstream processing, and regulatory compliance within scalable production architectures.

Despite these advantages, industrial and techno-economic assessments of microalgae remain largely biomass-centric, with limited emphasis on targeted metabolite generation, rational strain optimization, and product-specific value creation (Rahman, 2020; Vuppaladadiyam et al., 2018). Such biomass-oriented analyses often prioritize volumetric productivity without sufficiently addressing pathway controllability, metabolic stability, or product-specific purification constraints. Recent comprehensive reviews, including (Rajpoot et al., 2025), continue to prioritize biofuel-oriented strategies, underscoring the need to reassess microalgae through an industrial biotechnology and high-value product lens. While existing literature provides a strong foundation for biomass production and harvesting, it offers comparatively limited critical synthesis of metabolite biosynthesis regulation, process optimization strategies, downstream constraints, regulatory readiness, and market integration (Sivaramakrishnan et al., 2022). Consequently, the field lacks a consolidated framework that explicitly links biological regulation with techno-economic feasibility and regulatory determinants of industrial translation.

Importantly, the transition from bulk biomass production to HVP-centered biomanufacturing requires more than empirical adjustment of environmental and nutritional variables. It necessitates quantitative understanding of metabolic flux distribution, stress-responsive pathway regulation, cultivation kinetics, and downstream compatibility constraints. Rather than relying solely on iterative optimization, predictive modelling and data-integrated analysis enable rational design strategies grounded in metabolic network behaviour and process performance (Briassoulis et al., 2020; Ferreira et al., 2025). In this context, systems biology encompassing integrative omics, metabolic modelling, and predictive process analysis provides a conceptual foundation for connecting cellular regulation with industrial-scale performance. However, systematic incorporation of these quantitative tools into product-oriented industrial evaluations remains fragmented within current review literature.

Accordingly, this review synthesizes and critically evaluates recent advances in cultivation, biosynthesis, extraction, and genetic engineering of microalgal HVPs within a systems-informed industrial biotechnology framework. Rather than presenting descriptive summaries of isolated technologies, the analysis emphasises functional interdependencies among metabolic regulation, process engineering, purification strategy, safety validation, and regulatory governance. Upstream biology, downstream processing, and regulatory considerations are therefore examined as interlinked determinants of industrial viability rather than discrete operational stages. In contrast to biomass-focused analyses, this review prioritizes HVP development, rational process optimization, safety assessment, and commercialization readiness across major microalgal product classes. By integrating biologically informed design principles with industrial performance metrics, this work seeks to bridge the persistent gap between laboratory-scale optimization and scalable bioindustrial deployment. Overall, the evidence indicates a field progressing toward industrial validation, yet still constrained by persistent challenges in techno-economics, regulatory harmonization, scale-up robustness, and cross-stage process coordination that must be addressed to enable broader commercial adoption.

**FIGURE 1:**
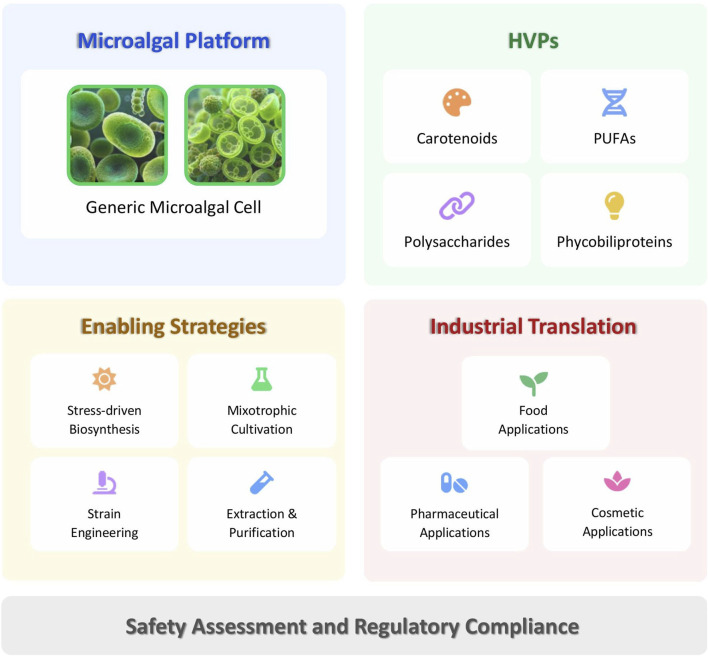
Microalgae as integrated biofactories for HVPs. A conceptual systems-level framework integrates current evidence, showing how major HVP classes interact with biological regulation, metabolic engineering, cultivation strategies, downstream processing, and regulatory governance. The framework highlights links among metabolic regulation, cultivation design, downstream processing, and safety assessment within an interconnected bioprocess system. It emphasises carbon flux control, process robustness, quality specification, and compliance as interdependent drivers of industrial optimization. Industrial translation to food, cosmetic, and pharmaceutical sectors requires rational optimization, scalability, techno-economic viability, and regulatory alignment.

Microalgae therefore represent more than alternative biomass sources; they function as programmable production platforms whose industrial relevance depends on strategic coordination among metabolic regulation, cultivation strategy, downstream processing, and regulatory compliance. This perspective reframes microalgal biotechnology as a systems-engineered production platform rather than a resource extraction paradigm. Figure 1 presents a proposed conceptual integrative framework, derived from and informed by current literature to illustrate how major microalgal HVP classes interface with enabling technologies and industrial translation pathways. Subsequent sections elaborate on each element of this framework, progressing from product classes to cultivation and strain-engineering strategies, safety assessment, regulatory considerations, and industrial deployment.

## 2 Microalgal HVPs: biological function, bioprocess integration, bio-industrial applications

**FIGURE 2:**
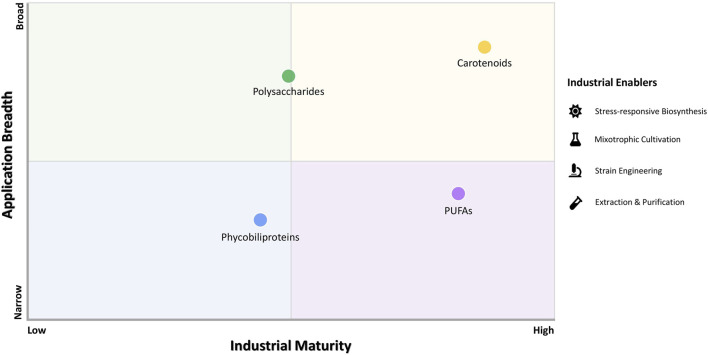
Industrial maturity and application readiness of major microalgal HVPs. A conceptual comparative matrix positions key HVP classes by industrial maturity, scalability, market penetration, and application breadth based on the analyzed studies discussed in this paper. Carotenoids are the most established, supported by well-characterized stress-responsive biosynthesis, scalable cultivation, and regulatory acceptance. PUFAs show high technological maturity and growing market integration but remain constrained by oxidative stability, purification selectivity, and cost competitiveness. Polysaccharides and phycobiliproteins remain emerging due to metabolic variability, integration challenges, structural heterogeneity, and purification complexity. Industrial readiness depends on systems-level integration of biosynthetic control, cultivation optimization, downstream compatibility, regulatory validation, and techno-economic feasibility.

Microalgae produce a diverse portfolio of HVPs that differ markedly in biological function, metabolic regulation, process maturity, and industrial readiness. These distinctions arise from variability in pathway controllability, metabolic flux stability, product recovery complexity, and regulatory classification. To contextualize this heterogeneity, Figure 2 presents a conceptually derived framework integrating evidence from the current literature to comparatively situate major microalgal HVP classes according to relative industrial maturity, scalability, and application breadth. This comparative assessment incorporates technological consolidation, downstream compatibility, and market integration dynamics alongside intrinsic biological attributes. This classification therefore reflects not only market presence but also the degree to which biosynthetic regulation, cultivation robustness, purification workflows, and regulatory feasibility have achieved industrial consolidation.

Within this framework, carotenoids represent the most industrially established class, followed by PUFAs, whereas polysaccharides and phycobiliproteins remain comparatively emerging. This maturity gradient corresponds to differences in metabolic predictability, oxidative stability, purification selectivity, and regulatory clarity. Such variation stems from disparities in metabolic stability, process coordination complexity, downstream selectivity requirements, and familiarity within regulatory jurisdictions. The following subsections therefore examine each HVP class in detail, integrating biological function, systems-informed bioprocess optimization, extraction constraints, techno-economic determinants, and market positioning to evaluate industrial feasibility.

### 2.1 Carotenoids: biological function and industrial relevance

Carotenoids are naturally occurring pigments synthesized by microalgae, plants, bacteria, and archaea, responsible for yellow, orange, or red coloration and light absorption between 450 and 550 nm. The carotenoid biosynthesis proceeds primarily through the methylerythritol phosphate (MEP) pathway within plastids, directly linking carbon assimilation to isoprenoid metabolism (Yabuzaki, 2017; Novoveská et al., 2019; Prates, 2025). Beyond their role in photosynthesis and photoprotection, carotenoids provide potent antioxidant activity, making them highly attractive for nutritional, cosmeceutical, and pharmaceutical industries. Structurally, they are divided into xanthophylls (oxygen-containing; e.g., astaxanthin, zeaxanthin) and carotenes (hydrocarbons; e.g., beta-carotene) (Novoveská et al., 2019; Prates, 2025). Microalgae produce unique carotenoids, including astaxanthin, fucoxanthin, canthaxanthin, and diatoxanthin, many of which are absent or rare in terrestrial plants or synthetic sources (Ambati et al., 2014; Takaichi, 2025; Mostafa and Hashem, 2024). This structural diversity facilitates targeted market positioning, as isomer composition, esterification patterns, and co-occurring metabolites influence functional performance, stability profiles, and regulatory classification. This diversity therefore enables metabolite-centric industrial strategies that link pigment production directly to commercial objectives, particularly in high-value markets where purity, natural origin, and functional bioactivity justify premium pricing.

Within microalgae, carotenoids exist as primary pigments integrated into photosynthetic membranes or as secondary carotenoids that accumulate under environmental stress such as high light intensity, nutrient limitation, or salinity, typically sequestered in lipid vesicles (Shah et al., 2025). The transition from primary to secondary carotenoid accumulation reflects a coordinated metabolic reprogramming involving redox modulation, energy redistribution, and transcriptional regulation of key biosynthetic enzymes. Moreover, the stress-responsive biosynthetic plasticity constitutes a biologically regulated control node that can be strategically exploited through informed bioprocess optimization. Rather than relying solely on empirical stress induction, emerging approaches increasingly combine metabolic pathway elucidation, transcriptomic and metabolomic profiling, and predictive modelling to rationally enhance carotenoid flux toward target compounds. Integration of multi-omics datasets with flux analysis supports quantitative identification of pathway bottlenecks and enables cultivation strategies grounded in measurable metabolic constraints. The physiological flexibility therefore provides a controllable mechanism for industrially relevant yield enhancement, directly connecting cellular regulation to cultivation design and downstream recovery considerations.

Their antioxidant, provitamin A, anti-inflammatory, and anticancer activities underpin their commercial value, offering functional advantages over plant-derived or synthetic pigments, including superior purity, batch consistency, and sustainability. However, industrial competitiveness depends not only on biological yield but also on volumetric productivity, extraction selectivity, oxidative stability, cost structure, and regulatory acceptance across target markets. Consequently, carotenoids represent the most consolidated microalgal HVP class because their biosynthetic regulation, cultivation optimization, and downstream processing have achieved comparatively advanced cross-stage coordination.

#### 2.1.1 Bioprocess optimization and stress-responsive biosynthesis

Industrial-scale carotenoid production requires precise coordination of environmental, nutritional, and biological variables to maximize yield without compromising cell viability. At the metabolic level, carotenoid accumulation represents a regulated carbon reallocation process in which flux is diverted from primary growth pathways toward secondary metabolite synthesis under defined stress conditions. This transition involves coordinated regulation of isoprenoid pathway enzymes, redox balance, and energy distribution within the chloroplast. Key stressors including light intensity and spectrum, temperature, and nutrient limitation activate enzymatic pathways that stimulate carotenoid biosynthesis (Zhao et al., 2025; Roldan-Prieto et al., 2024; Papapanagiotou et al., 2024). For example, *Dunaliella salina* can accumulate beta-carotene up to 50.6% under nitrogen limitation, with red LED illumination further enhancing production, whereas lutein content in *Dunaliella* sp. increases by 95.9% under optimized light conditions (Wu et al., 2020). Similarly, *Haematococcus pluvialis* astaxanthin productivity rises by 50% under combined red and blue light at 30 °C (Pereira and Otero, 2020).

While such empirical optimization strategies demonstrate substantial yield improvements, their industrial translation depends on predictive control of metabolic flux distribution, stress tolerance thresholds, and cultivation robustness. Reliance on empirical stress induction alone may introduce variability in pigment composition, productivity instability, and limited reproducibility under large-scale photobioreactor conditions. Recent advances in systems biology including transcriptomic profiling, proteomic analysis, metabolic flux modeling, and genome-scale network reconstruction enable identification of regulatory bottlenecks and key enzymatic control nodes governing carotenoid biosynthesis. Coupling multi-omics datasets with quantitative flux analysis supports simulation of carbon allocation under defined environmental scenarios, enabling predictive process design rather than reactive adjustment. These approaches facilitate rational strain design and targeted pathway amplification, reducing dependence on trial-and-error optimization.

Multi-factor strategies including co-cultivation, metal ion supplementation, and chemical elicitors such as ethanol or GABA consistently outperform single-variable approaches, demonstrating the value of coordinated bioprocess interventions (Lu et al., 2019). However, industrial feasibility requires balancing enhanced pigment accumulation with productivity stability, energy consumption, and downstream compatibility. Economic viability further depends on light-use efficiency, photobioreactor configuration, mixing energy demand, and alignment with harvesting and extraction workflows. When coupled with LED-controlled photobioreactors and genome-scale metabolic engineering, such multi-dimensional optimization enables scalable and economically viable carotenoid production (Zhang et al., 2020). The convergence of stress-responsive cultivation, model-informed metabolic engineering, and controlled photobioreactor design exemplifies a rational biorefinery strategy in which upstream regulation, process parameters, techno-economic constraints, and downstream recovery are addressed through coordinated design rather than isolated variable adjustment.

#### 2.1.2 Extraction technologies and industrial bottlenecks

Carotenoid extraction remains a major techno-economic bottleneck in microalgal production, often representing up to 90% of total production costs and significantly influencing overall process scalability (Zhang et al., 2020; Patel, 2025). This constraint reflects not only extraction inefficiency but also its substantial contribution to capital expenditure, operational costs, and final product pricing. This bottleneck arises not only from solvent requirements but also from cell wall recalcitrance, pigment localization within lipid bodies, energy-intensive biomass drying, and the need to preserve oxidative stability during recovery. Conventional solvent-based methods employing hexane, acetone, or ethanol are widely used at laboratory and industrial scales due to their simplicity and high extraction efficiency. However, their continued industrial deployment is increasingly constrained by solvent toxicity, residual solvent limits, worker safety regulations, environmental compliance costs, and downstream purification requirements, particularly in food, nutraceutical, and cosmeceutical applications (Routray et al., 2019; Yusof et al., 2025). These constraints are even more stringent in pharmaceutical-grade production, where impurity thresholds and solvent residue specifications are tightly regulated.

To overcome these limitations, advanced extraction strategies have been developed. Enzyme-assisted extraction facilitates degradation of robust microalgal cell walls and reduces solvent consumption; when coupled with ultrasonication, it can achieve astaxanthin recovery up to 83.9% (Machado et al., 2016; Nadar et al., 2018). Supercritical ${\text{CO}}_{2}$ extraction (SFE) provides a mild, solvent-free process capable of high-purity carotenoid recovery, reaching 98.6% for astaxanthin and 52.3% for lutein (Khoo et al., 2019; Yousefi et al., 2019). The selectivity of SFE can be further tuned through pressure modulation and co-solvent addition, enabling targeted recovery of specific carotenoid fractions. Hybrid microwave- and ultrasonic-assisted methods further enhance extraction efficiency and reduce processing time (Alpural et al., 2024; Mishra et al., 2025). Nevertheless, these techniques require precise thermal and process control to prevent degradation and isomerization of sensitive carotenoid structures.

Despite these technical advantages, large-scale implementation of green and hybrid extraction technologies remains limited. High capital investment, specialised equipment requirements, parameter sensitivity, scale-up complexity, and energy demand often offset laboratory-scale performance gains. Moreover, alignment with upstream cultivation strategies and downstream formulation workflows is not yet standardized across microalgal species, creating additional barriers to industrial deployment. Variability in cell wall composition, pigment distribution, and biomass moisture content further complicates process transferability between strains.

Comparative analysis therefore indicates that while green and hybrid strategies offer improved environmental profiles and product purity, their economic competitiveness depends on coordinated optimization across cultivation, biomass handling, extraction design, and regulatory compliance (Sharma et al., 2025; Paz et al., 2024). Extraction efficiency cannot be optimized in isolation; carotenoid stability, cell wall architecture, stress-induced pigment accumulation, and moisture content function as interdependent process variables. Process intensification strategies, including wet extraction workflows and *in situ* product recovery, are increasingly explored to reduce drying energy demand and enhance overall process performance. Rational process design therefore requires aligning stress-responsive biosynthesis, harvest timing, and extraction modality within a unified biorefinery strategy. Such coordinated design improves economic feasibility, regulatory robustness, batch consistency, and long-term product stability, reinforcing the strategic potential of microalgae as scalable and sustainable platforms for high-value carotenoid production.

#### 2.1.3 Bioindustrial applications and market integration of microalgal carotenoids

Microalgal carotenoids constitute one of the most industrially advanced classes of HVPs, particularly within nutritional, cosmeceutical, and emerging pharmaceutical markets. Recent industry market analyses estimate that the global carotenoids market was valued at approximately USD 2.1 billion in 2024 and is projected to reach nearly USD 2.9 billion by 2029, reflecting a compound annual growth rate (CAGR) of approximately 6.7% (Research and Markets, 2025). This sustained expansion aligns with peer-reviewed assessments describing increasing industrial consolidation of natural microalgal pigments and growing demand for high-purity, sustainably sourced antioxidant compounds (Razzak, 2024). Within this landscape, natural astaxanthin derived predominantly from *H. pluvialis* represents one of the most commercially established premium segments. Commercial-scale production of astaxanthin from *H. pluvialis* and beta-carotene from *D. salina* exemplifies technological maturity, process scalability, and regulatory feasibility of carotenoid-focused biomanufacturing platforms, supported by strain optimization, stress-responsive biosynthesis control, and robust downstream purification workflows.

In nutritional applications, beta-carotene, lutein, and astaxanthin support visual health, immune modulation, and oxidative stress mitigation (Sathasivam et al., 2019; Martínez-Ruiz et al., 2025; Dhandwal et al., 2025). Their incorporation into dietary supplements and fortified formulations reflects established regulatory acceptance, including GRAS status in specific jurisdictions. Compared with synthetic carotenoids, microalgal-derived pigments provide intrinsic antioxidant bioactivity and defined stereochemical profiles. This stereochemical specificity, particularly in astaxanthin isomer distribution, contributes to enhanced biological efficacy and supports premium positioning in nutraceutical and clinical-grade markets. In cosmeceutical applications, carotenoids such as astaxanthin and fucoxanthin are incorporated into anti-aging and photoprotective formulations (Adamantidi et al., 2025; Najafi et al., 2025). However, susceptibility to oxidative degradation necessitates encapsulation strategies, antioxidant co-formulation, and stability validation protocols to ensure consistent bioactivity during storage and application.

Within pharmaceutical research contexts, carotenoids are investigated for anti-inflammatory and cardioprotective properties (Shanaida et al., 2025; Sarkar and Ray, 2025). Progression toward regulated pharmaceutical deployment requires reproducible isomer composition, impurity profiling, pharmacokinetic characterization, and compliance with advanced regulatory frameworks governing active ingredients. Collectively, the commercial consolidation of carotenoids reflects coordinated alignment of metabolic regulation, cultivation strategy, purification control, quality specification, and regulatory validation. This maturity positions carotenoids as a reference model for translational advancement of other microalgal HVPs.

### 2.2 PUFAs: biological function and industrial relevance

Polyunsaturated fatty acids (PUFAs), particularly long-chain omega-3s such as eicosapentaenoic acid (EPA) and docosahexaenoic acid (DHA), are essential bioactive lipids supporting cardiovascular function, neurodevelopment, immune modulation, and inflammatory regulation (Saini and Keum, 2018; Bagchi et al., 2025). Their roles in membrane fluidity modulation, eicosanoid signalling cascades, and neuroprotective pathways underpin their therapeutic and functional relevance across nutritional, clinical, and cosmetic applications. Their established significance in clinical nutrition, pharmaceutical research, and dermatological formulations underscores their cross-sector importance. Conventional omega-3 supply remains heavily dependent on marine fisheries, which are increasingly constrained by environmental variability, contamination risks, and sustainability concerns (Zheng et al., 2025). Such dependency introduces supply-chain volatility and limits traceability, particularly in high-purity medical and infant nutrition markets.

Microalgae, as primary producers of marine omega-3s, provide a controlled and traceable route for EPA and DHA production (Ryckebosch et al., 2014; Saini and Keum, 2018; Abera and Adimas, 2024). Taxa such as Bacillariophyta, Chlorophyta, and Heterokontophyta naturally accumulate high omega-3 content (Ryckebosch et al., 2014). Unlike fish-derived oils, microalgal lipids are generated under tightly regulated cultivation environments that enable specification-driven fatty acid profiling and systematic contaminant monitoring. Compared with fisheries-dependent supply chains, such controlled production supports defined fatty acid composition, reduced pollutant exposure, and improved batch consistency attributes particularly relevant for pharmaceutical and cosmeceutical applications.

Genera including *Nannochloropsis*, *Schizochytrium*, and *Chlorella* have demonstrated scalable EPA and DHA production capacities (Sun et al., 2024; Wang P. et al., 2024). However, interspecies variability in elongation and desaturation pathways, lipid storage architecture, and oxidative susceptibility influences yield stability, process design, and downstream purification requirements. Industrial relevance therefore depends not solely on lipid productivity but also on coordinated control of metabolic flux distribution, cultivation parameters, oxidative stability, and purification selectivity. Effective deployment requires strategic alignment of strain selection, metabolic regulation, reactor configuration, harvest timing, and regulatory purity specifications within a coherent production architecture. Microalgae thus function as programmable lipid biofactories whose commercial translation depends on synchronising biological performance with process engineering and regulatory compliance.

#### 2.2.1 Bioprocess optimization and stress-responsive biosynthesis

PUFA accumulation in microalgae is strongly influenced by environmental and metabolic regulation, with controlled stress commonly employed to enhance lipid production (D’Alessandro and Antoniosi Filho, 2016; Song et al., 2024; Dubey et al., 2024). At the cellular level, nutrient limitation induces metabolic reallocation from protein synthesis toward lipid biosynthesis, redirecting carbon flux and reducing nitrogen-intensive anabolic pathways. Nitrogen or phosphorus deprivation can significantly increase lipid content in species such as *Nannochloropsis oculata* and *Chlorella pyrenoidosa*, although this frequently reduces overall biomass productivity (Rasdi and Qin, 2015; Abrha et al., 2025; Abdelkarim et al., 2025). Two-stage cultivation strategies, initial nutrient-replete growth followed by controlled nutrient starvation, offer a practical industrial compromise, balancing biomass accumulation with enhanced lipid yield (Mathimani et al., 2018; Dubey et al., 2024).

While stress-induced lipid enhancement demonstrates clear laboratory-scale effectiveness, reliance on empirical stress application alone often results in productivity trade-offs, metabolic instability, and scale-up unpredictability. Such empirical approaches lack predictive resolution and may generate variable fatty acid profiles under fluctuating industrial conditions. Species-specific physiological responses further complicate optimization: EPA is predominantly synthesized in Chlorophyceae and Cryptophyceae, whereas DHA preferentially accumulates in dinoflagellates and *Schizochytrium* (Chauton et al., 2015; Remize et al., 2021; Eltanahy and Torky, 2021; Soto-Sánchez et al., 2023). Additional stressors including high light, salinity, and temperature shifts modulate PUFA profiles but may simultaneously suppress growth or alter fatty acid composition unpredictably Maltsev et al. (2023); Suparmaniam et al. (2023).

Recent advances in systems biology provide tools to address these limitations. Transcriptomic, proteomic, and metabolomic analyses enable identification of regulatory bottlenecks and key enzymes governing fatty acid elongation and desaturation pathways. Coupling multi-omics datasets with genome-scale metabolic modelling and flux balance analysis allows quantitative prediction of carbon partitioning under defined cultivation scenarios. These computational approaches support predictive optimization of lipid accumulation while preserving cellular viability and metabolic stability. Such frameworks enable rational strain engineering, dynamic pathway modulation, and data-informed adjustment of cultivation parameters, thereby reducing reliance on trial-and-error stress regimes.

From an industrial perspective, effective PUFA bioprocess design requires coordinated control of metabolic regulation, cultivation strategy, harvest timing, oxidative stability management, and purification selectivity. Rather than treating stress induction, strain engineering, and reactor configuration as isolated interventions, predictive modelling frameworks align upstream physiological responses with engineering constraints, regulatory purity specifications, and techno-economic performance targets. This multi-dimensional strategy provides a more robust pathway toward scalable, high-purity, and economically viable microalgal omega-3 production.

#### 2.2.2 Extraction technologies and industrial bottlenecks

Robust microalgal cell walls constitute a primary bottleneck for PUFA extraction, influencing lipid accessibility, solvent penetration, energy demand, and final product purity (Cuellar-Bermudez et al., 2015; Zhou et al., 2022; Shivakumar et al., 2024). Beyond mechanical resistance, intracellular compartmentalization of triacylglycerols and the high susceptibility of long-chain PUFAs to oxidative degradation further constrain efficient downstream recovery. Conventional solvent-based methods, including hexane extraction, remain widely applied due to operational simplicity and established industrial familiarity. However, these approaches present contamination risks, require multiple refining steps, and offer limited selectivity for highly unsaturated fatty acids (Motlagh et al., 2024; Costa et al., 2024). Residual solvent limits, regulatory compliance requirements, and oxidative instability during processing impose additional constraints, particularly for pharmaceutical- and cosmeceutical-grade omega-3 products where purity specifications are stringent.

Multi-step purification workflows such as fractional and molecular distillation can concentrate EPA and DHA fractions, yet these processes increase operational complexity, energy consumption, and capital investment, thereby reducing economic competitiveness at large scale Yan et al. (2025); Barkia et al. (2019). Excessive thermal exposure during purification may compromise PUFA structural integrity and bioactivity, necessitating strict temperature control and antioxidant stabilisation strategies.

Emerging techniques including ultrasonication, microwave-assisted extraction, Soxhlet extraction, and supercritical ${\text{CO}}_{2}$ extraction (SFE) aim to enhance lipid recovery while reducing solvent dependence (Mathimani et al., 2017; 2018; Quesada-Salas et al., 2021; Mishra et al., 2025). Ultrasonication and microwave-assisted methods improve cell disruption efficiency but may induce localized heating and lipid oxidation if not carefully controlled. Soxhlet extraction remains solvent-intensive and energy-demanding. SFE offers high-purity, solvent-free recovery under relatively mild conditions and is compatible with food and pharmaceutical regulatory standards. Nevertheless, SFE performance remains sensitive to moisture content, pressure optimization, co-solvent selection, and feedstock pre-treatment, all of which influence selectivity and yield.

Despite these advantages, widespread industrial adoption of green extraction technologies remains limited by high capital expenditure, specialized equipment requirements, process parameter sensitivity, and scale-up complexity. Economic feasibility therefore depends on coordinated control of upstream lipid accumulation, moisture management, cell disruption efficiency, extraction selectivity, oxidative stabilisation, and downstream purification compatibility. Extraction performance cannot be considered independently of cultivation strategy, harvest timing, and fatty acid profile management, as these variables collectively determine yield stability and product quality.

Consequently, the most promising pathway for industrial PUFA production combines stress-informed lipid enhancement, strain engineering for defined fatty acid profiles, and green extraction platforms such as SFE within a coherent biorefinery strategy. This alignment of metabolic regulation, process selectivity, regulatory purity requirements, and techno-economic constraints strengthens industrial robustness and scalability while reducing solvent reliance and enhancing product quality for nutritional, cosmetic, and pharmaceutical applications.

#### 2.2.3 Bioindustrial applications and market integration of microalgal PUFAs

Microalgal long-chain omega-3 PUFAs, particularly EPA and DHA, are increasingly positioned as strategic alternatives to fish-derived oils due to enhanced traceability and controlled production conditions (Ryckebosch et al., 2014; Saini and Keum, 2018; Rathinavel et al., 2025). Industry analyses estimate that the algae-derived omega-3 ingredients market was valued at approximately USD 1.56 billion in 2026 and is projected to reach nearly USD 2.73 billion by 2031 (CAGR 11.74%) (Mordor Intelligence, 2024). Broader algae-based omega-3 production systems were valued at approximately USD 3.8 billion in 2024, with projections exceeding USD 20 billion by 2034, reflecting sustained double-digit growth trajectories (The Business Research Company, 2025). These figures indicate accelerating commercial expansion of microalgal lipid platforms. These market projections align with peer-reviewed analyses highlighting structural vulnerabilities in fisheries-dependent supply chains and the strategic role of microalgae as scalable primary omega-3 producers (Saini and Keum, 2018). Microalgal production enables year-round manufacturing with defined fatty acid profiles and batch consistency, attributes critical for clinical and infant nutrition formulations. The absence of heavy metal accumulation and persistent pollutants further addresses regulatory and safety concerns. Nevertheless, pharmaceutical-grade deployment requires rigorous standardization, impurity profiling, and specification control.

In nutritional applications, microalgal EPA and DHA are incorporated into supplements and fortified products (Dhandwal et al., 2025; Santin et al., 2022). Although fish oil continues to dominate bulk commodity markets, microalgal-derived products are consolidating premium segments characterized by higher purity standards, traceability requirements, and sustainability-driven procurement strategies. In cosmetic applications, EPA- and DHA-rich oils contribute to membrane repair and anti-inflammatory formulations (De Luca et al., 2021; Prates, 2025; Lionti, 2024). Their susceptibility to oxidative degradation necessitates encapsulation technologies and controlled stabilization strategies to preserve lipid integrity during storage and application.

From a pharmaceutical perspective, purified EPA and DHA are under investigation for cardioprotective and neuroprotective applications (Ma et al., 2025; Afroze et al., 2024; Kariyawasam et al., 2025). Regulatory progression toward fully approved therapeutic products requires dose standardization, bioavailability validation, oxidative stability control, and compliance with pharmaceutical manufacturing frameworks. Overall, microalgal EPA and DHA represent commercially advanced HVP categories supported by scalable cultivation, defined lipid profiling, and progressive regulatory validation. Their maturation reflects convergent optimization of metabolic engineering, cultivation strategy, purification control, quality specification, and regulatory compliance within coordinated production systems.

### 2.3 Polysaccharides and phycobiliproteins as emerging microalgal HVPs

Beyond carotenoids and PUFAs, microalgae synthesize structurally diverse polysaccharides and phycobiliproteins that are gaining attention for applications in food, cosmetic, and biopharmaceutical sectors (Eladl et al., 2024; Minić et al., 2024). Compared with more industrially consolidated metabolite classes, these compounds exhibit greater compositional variability and less predictable process performance. Unlike carotenoids and omega-3 lipids, these products remain at earlier stages of industrial development, largely due to variability in yield, structural heterogeneity, purification complexity, and limited regulatory harmonization. Nevertheless, their multifunctional bioactivity and compatibility with high-value niche markets position them as promising candidates within diversified microalgal biorefinery platforms.

Microalgal polysaccharides can constitute approximately 10%–52% of biomass and exhibit antioxidant, anticoagulant, antiviral, and anti-inflammatory activities (Mehariya et al., 2021; Olguín et al., 2022). Species such as *Porphyridium* spp. are particularly attractive due to their secretion of extracellular polysaccharides (EPS), enabling partial decoupling of product recovery from cell disruption (Kavitha et al., 2016; Li et al., 2019). This secretion-based strategy reduces mechanical processing requirements but shifts process control challenges toward culture rheology management, mass transfer efficiency, and purification selectivity. Sulfated polysaccharides from *Porphyridium cruentum* demonstrate antiviral and antitumor activities (Huheihel et al., 2002; Gardeva et al., 2009), supporting pharmaceutical interest; however, clinical translation necessitates rigorous structural standardization, molecular weight control, reproducibility of sulfation patterns, pharmacokinetic evaluation, and comprehensive toxicological validation.

In food and cosmetic formulations, microalgal polysaccharides function as natural thickeners, stabilizers, and moisture-retention agents, contributing to texture enhancement and barrier reinforcement. Compared with terrestrial hydrocolloids, microalgal polysaccharides offer renewable production routes compatible with saline and wastewater cultivation systems, enhancing sustainability. Industrial competitiveness, however, depends on improving volumetric productivity, mitigating viscosity-induced mixing limitations, and ensuring batch-to-batch compositional consistency under variable cultivation conditions.

Phycobiliproteins represent a second emerging HVP class, predominantly derived from red microalgae such as *Porphyridium* spp. and *Porphyra haitanensis*. Pigments including phycoerythrin and phycocyanin exhibit antioxidant, anti-inflammatory, immunomodulatory, and anticancer activities (Pan et al., 2013; Gargouch et al., 2018). Their intense coloration also supports applications in natural food colorants and cosmetic formulations. However, phycobiliproteins are highly sensitive to light, pH, and temperature fluctuations, resulting in stability constraints during extraction, purification, and formulation. This physicochemical sensitivity necessitates controlled processing environments and stabilisation strategies, including encapsulation or antioxidant co-formulation, to preserve bioactivity and shelf stability. In addition, intracellular localization requires efficient yet gentle cell disruption methods to maintain structural integrity.

Advancing polysaccharides and phycobiliproteins toward industrial maturity requires coordinated control of strain selection, secretion efficiency, cultivation rheology, product stability, and downstream recovery. Rather than sequential optimization of isolated process steps, cohesive design strategies linking metabolic regulation, secretion pathways, reactor configuration, purification workflows, and regulatory validation are needed to overcome current bottlenecks. While these compounds remain less mature than carotenoids and PUFAs, their expanding functional applications and compatibility with high-value markets justify continued development within next-generation microalgal biorefinery systems.

## 3 Integrated cultivation and strain engineering strategies for microalgal HVP production

The industrial production of HVPs including carotenoids, PUFAs, and polysaccharides remains constrained by limited metabolic plasticity and suboptimal productivity in conventional photoautotrophic and heterotrophic cultivation systems (Ruiz et al., 2016). Photoautotrophic cultures, typically operated in open ponds or photobioreactors, are restricted by light attenuation, fluctuating irradiance, contamination risk, and high energy demand for mixing and temperature control, while heterotrophic systems depend on costly organic substrates and cannot exploit light-induced secondary metabolite synthesis (Patel et al., 2021; Deschamps et al., 2008). Although both systems can achieve substantial biomass accumulation, neither inherently directs carbon partitioning and metabolic flux allocation toward specific high-value metabolites. As a result, traditional cultivation modes often favour growth over selective HVP biosynthesis.

Mixotrophic cultivation partially addresses these constraints by integrating photosynthetic carbon fixation with organic carbon assimilation, enabling simultaneous operation of respiratory and photosynthetic pathways (Pang et al., 2019; Yeh and Chang, 2012). Species such as *Chlorella* sp., *Haematococcus pluvialis*, and *Botryococcus braunii* exhibit enhanced growth rates and improved HVP yields under mixotrophic conditions, particularly when low-cost substrates or wastewater streams are utilised (Li et al., 2016; Pang and Chen, 2017). At the metabolic network level, mixotrophy expands redox buffering capacity and energy availability, enabling more flexible redistribution of carbon flux toward target metabolites. Nevertheless, performance remains highly strain-dependent and sensitive to substrate concentration, light intensity, and nutrient balance, resulting in variability in productivity and complicating process scale-up, reproducibility, and techno-economic predictability.

Genetic engineering and gene-editing approaches further strengthen industrial performance by enabling targeted redirection of metabolic flux toward desired HVP pathways. Advances in CRISPR-based editing, transcriptomic profiling, proteomic analysis, and genome-scale metabolic modelling have enabled identification of rate-limiting enzymes, regulatory nodes, and flux bottlenecks governing carotenoid and PUFA biosynthesis. Targeted modifications in species such as *H. pluvialis*, *Phaeodactylum tricornutum*, and *Nannochloropsis oceanica* have increased product yields while enhancing photosynthetic efficiency, stress tolerance, and growth robustness (Galarza et al., 2018; Hamilton et al., 2014; Osorio et al., 2019).

Beyond yield enhancement, engineered traits that reduce cell wall recalcitrance, improve nutrient uptake efficiency, optimize carbon allocation, and stabilise metabolite accumulation directly support downstream processing efficiency and regulatory compliance (Ahmed et al., 2024; Chuang et al., 2025). These modifications also enhance process stability, batch reproducibility, and compatibility with large-scale cultivation platforms. However, industrial translation of genetically modified strains remains influenced by regulatory frameworks, public acceptance considerations, and containment requirements, particularly in food and cosmetic markets. Regulatory approval pathways for engineered microalgae are typically more stringent than for conventional strains, requiring comprehensive molecular characterization, genetic stability assessment, and risk-based environmental evaluation. Table 1 synthesises the biological, technical, and regulatory determinants that govern the feasibility and industrial translatability of engineered microalgal strains.

**TABLE 1:** Key determinants governing the industrial translation of genetic engineering strategies in microalgae. The table summarizes the biological, technical, and regulatory factors that collectively determine the scalability, genetic stability, and commercial feasibility of engineered microalgal strains for HVP production. These determinants including metabolic performance, long-term genetic integrity, cultivation compatibility, downstream process integration, and regulatory compliance requirements operate as interconnected constraints within a systems-level industrial optimization framework.

Determinant	Role in HVP strain development	Impact on industrial performance	Translation constraints
Species selection	Selection of microalgal species with inherent capacity for target HVP accumulation (e.g., *Haematococcus pluvialis* for astaxanthin; *Phaeodactylum tricornutum* for PUFAs)	Strong influence on achievable yields, baseline productivity, and process robustness	Limited number of industrially tractable species; domestication and scale-up bottlenecks
Target gene identification	Manipulation of key enzymes within carotenoid and PUFA biosynthetic pathways (e.g., *pds*, *psy*, Δ5 -elongase)	Enables substantial enhancement of product titers and improved control of metabolic flux	Requires detailed pathway knowledge; risk of metabolic imbalance and growth penalties
Transformation strategy	Delivery of genetic constructs via biolistics, electroporation, or emerging CRISPR-based systems	Determines feasibility, efficiency, and reproducibility of strain construction across taxa	Species-dependent success rates; limited molecular toolkits for many industrial strains
Stability of gene expression	Maintenance of long-term, heritable transgene expression under industrial cultivation conditions	Critical for consistent HVP productivity during prolonged cultivation and scale-up	Transgene silencing, genetic instability, and loss of expression over time
Post-engineering cultivation optimization	Alignment of engineered metabolism with light regimes, nutrient availability, and stress induction strategies	Strong synergistic effects on final HVP yields and process efficiency	Requires tight process control; increased operational and scale-up complexity
Regulatory and biosafety compliance	Assessment of GMO status, containment requirements, and product approval pathways	Determines market accessibility, particularly for food, cosmetic, and pharmaceutical applications	Stringent regulatory frameworks restrict outdoor cultivation and delay commercialization

**FIGURE 3:**
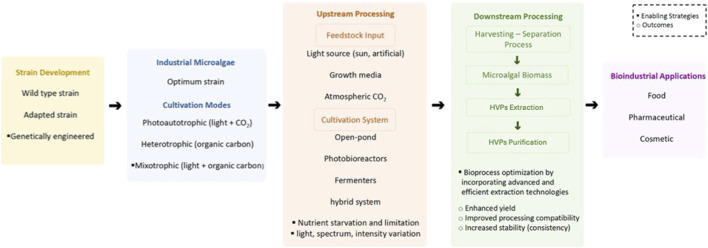
Integrated bioprocess framework for microalgal HVP production and industrial translation. Conceptual systems-level schematic illustrating the coordinated integration of strain development, metabolic engineering, and cultivation strategy to regulate carbon flux distribution and stress-responsive biosynthesis of target HVPs. The framework further integrates harvesting, extraction, purification, and stabilization steps to ensure alignment between upstream metabolic regulation and downstream process compatibility, product quality attributes, and regulatory compliance requirements. This coordinated design approach emphasizes rational, predictive optimization across the entire value chain to enhance scalability, techno-economic feasibility, process robustness, and industrial deployment readiness.

Overall, effective HVP production depends on strategic alignment of cultivation strategy, strain engineering, metabolic regulation, and downstream constraints within a coherent optimization architecture. Rather than sequentially refining isolated process components, predictive modelling and data-integrated design align carbon flux control, cultivation mode selection, reactor configuration, harvest timing, extraction compatibility, and regulatory validation within a coordinated decision framework. This approach enables microalgae to function as programmable biofactories capable of delivering scalable, economically viable, and regulatory-compliant HVP production across food, cosmetic, and biopharmaceutical sectors. Figure 3 illustrates this coordinated bioprocess perspective, highlighting how strain development, cultivation design, and downstream considerations collectively determine industrial feasibility.

## 4 Risk assessment and regulatory aspects of microalgal HVPs

The expanding application of microalgal high-value products (HVPs) across food, cosmetic, and pharmaceutical sectors necessitates structured and product-specific safety evaluation to ensure consumer protection and regulatory compliance. Such evaluation must be proportionate to the intended use, exposure route, and population group, particularly where systemic exposure or long-term consumption is anticipated. Although microalgae provide controlled and traceable production systems relative to terrestrial or marine sources, their strain-specific metabolic diversity and cultivation-dependent variability introduce distinct compositional and regulatory considerations that must be systematically addressed.

**FIGURE 4:**
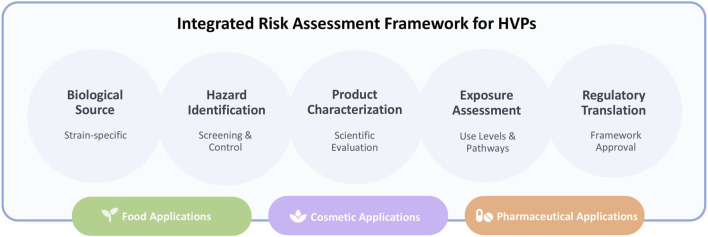
Integrated risk assessment and regulatory framework for microalgal HVPs. Conceptual systems-level schematic illustrating the coordinated alignment of strain identification, hazard screening, compositional characterization, and exposure assessment with regulatory evaluation pathways across food, cosmetic, and pharmaceutical sectors. The framework emphasizes traceability, controlled production conditions, and evidence-based safety validation as interconnected determinants of scalable, compliant, and risk-proportionate industrial deployment.

As microalgal HVPs transition from niche applications to scalable industrial products, risk assessment frameworks must integrate hazard identification, product characterisation, exposure context, and regulatory classification in a coherent manner. This integrated approach ensures that biological variability, processing conditions, and formulation characteristics are coherently reflected in regulatory decision-making and safety validation requirements. Figure 4 summarises this integrated regulatory logic, illustrating how strain attributes, compositional profiling, and intended market use collectively determine the level and type of safety validation required across food, cosmetic, and pharmaceutical domains.

### 4.1 Risk assessment

#### 4.1.1 Microalgal HVPs identification and hazard screening

Accurate taxonomic and strain-level identification constitutes the first critical step in the safety evaluation of microalgal HVPs, as closely related taxa may differ substantially in metabolic potential and toxicity profiles. Although widely commercialised strains such as *Chlorella vulgaris* and *Haematococcus pluvialis* are established producers of carotenoids and omega-3 lipids with documented safety records, other taxa including *Aphanizomenon flos-aquae* and *Microcystis aeruginosa* are capable of producing cyanotoxins such as microcystins with hepatotoxic and neurotoxic effects (Turck et al., 2025; Ampofo and Abbey, 2022). This divergence highlights the necessity of strain-level verification, metabolic profiling, and toxigenic screening rather than reliance on genus-level classification, particularly where products are intended for ingestion or systemic exposure.

Modern regulatory oversight increasingly requires molecular authentication tools, including DNA barcoding, strain fingerprinting, and whole-genome sequencing, to confirm taxonomic identity, genetic stability, and absence of toxin biosynthesis pathways. These approaches support traceability across cultivation batches and reduce the risk of inadvertent strain substitution or contamination with toxigenic variants. In the European Union, the European Food Safety Authority (EFSA) conducts safety assessments for food and feed applications under the Novel Food framework, while pharmaceutical and cosmetic uses are regulated under sector-specific legislation. In Saudi Arabia, the Saudi Food and Drug Authority (SFDA) oversees microalgal-derived products for food, cosmetic, and pharmaceutical markets through harmonised but application-dependent regulatory pathways. In the United States, the Food and Drug Administration (FDA) regulates food and dietary supplement ingredients under GRAS and dietary supplement provisions, while cosmetics and pharmaceuticals are evaluated under distinct sections of the Federal Food, Drug, and Cosmetic Act (FD&C Act). Despite jurisdictional differences, regulatory convergence is evident in the emphasis on documented strain traceability, production consistency, and risk-based safety characterisation tailored to intended use (EU2, 2015; COGEM, 2021).

Beyond intrinsic strain properties, cultivation conditions and production inputs represent critical hazard control points. The use of saline water, recycled media, or wastewater-derived nutrients can enhance sustainability but may introduce heavy metals, organic contaminants, pathogenic microorganisms, or residual toxins. Risk magnitude is therefore influenced not only by strain genetics but also by cultivation environment, harvesting strategy, and downstream purification efficiency. Regulatory tolerance for such inputs varies by sector, with the most stringent requirements typically applied to food and pharmaceutical applications involving systemic exposure.

Comprehensive analytical monitoring including ICP-MS for elemental contaminants and LC–MS or GC–MS for organic residues, toxins, and secondary metabolites is essential to demonstrate compliance and batch consistency. In this context, closed or semi-closed photobioreactor systems offer enhanced contamination control, environmental isolation, and reproducibility compared with open ponds (Fang et al., 2024). From a systems-level perspective, effective hazard screening integrates molecular strain authentication, cultivation control, environmental monitoring, and analytical verification within a unified quality management framework. Such integration is fundamental to ensuring that microalgal HVP production remains both scalable and regulatory compliant across diverse end-use markets.

#### 4.1.2 Product characterization and scientific evaluation

Comprehensive characterization of microalgal HVPs is central to their safe deployment across food, cosmetic, and pharmaceutical sectors and extends beyond compositional analysis to include toxicological, allergenicity, and exposure-based evaluation. Robust product characterization must ensure not only identity and purity, but also structural integrity, stability under processing conditions, and reproducibility across production batches. Analytical techniques such as high-performance liquid chromatography (HPLC), liquid chromatography–mass spectrometry (LC–MS), and spectroscopic methods are routinely applied to quantify proteins, PUFAs, carotenoids, and other bioactive constituents, while ensuring batch consistency and stability across processing and formulation stages (Anaemene et al., 2025; Martín-Girela et al., 2020). For emerging or non-conventional species, extended profiling may be required to identify minor secondary metabolites, degradation products, or unexpected bioactive compounds that could influence safety assessment outcomes. For food and feed applications, additional screening for antinutritional factors and contaminants remains essential to confirm nutritional suitability and compliance.

Toxicological evaluation generally follows a tiered risk-based approach adaptable across sectors. Initial *in vitro* assays (e.g., Ames and micronucleus tests) are used to assess genotoxic potential, while cytotoxicity testing using relevant human or mammalian cell lines (e.g., hepatocyte, intestinal epithelial, or keratinocyte models) can provide early insight into dose-dependent cellular responses. These are followed by subchronic and chronic *in vivo* studies where required. For novel microalgal strains or purified HVP fractions intended for systemic exposure, additional endpoints including reproductive toxicity, developmental toxicity, and immunotoxicity may be necessary to meet pharmaceutical-level regulatory standards. Available evidence indicates that widely commercialized products such as *Spirulina* and *Chlorella* exhibit high safety margins, with no adverse effects observed at exposure levels well above typical dietary intake. Similarly, astaxanthin and omega-3 PUFAs derived from *H. pluvialis* and *Nannochloropsis* species show no evidence of carcinogenicity or reproductive toxicity at recommended doses, supporting their use in nutritional, cosmetic, and pharmaceutical formulations (Anaemene et al., 2025; Cavalletti, 2025).

Allergenicity assessment is particularly relevant for food and nutraceutical applications but also informs cosmetic safety, especially for leave-on products. In silico and *in vitro* IgE-binding studies indicate a generally low allergenic potential for most commercial microalgal strains, although precautionary labeling and post-market surveillance are recommended for sensitive populations (Cruz and Vasconcelos, 2024). Protein profiling and digestibility analyses further support allergenicity evaluation by identifying potential cross-reactive epitopes. Compared with terrestrial protein sources, microalgal HVPs typically display favorable allergenicity profiles, especially when appropriate processing steps such as heat treatment or enzymatic hydrolysis are applied.

Across sectors, exposure route and use pattern determine the scope of evaluation. Oral exposure dominates food and nutraceutical applications, dermal exposure is central for cosmetics, and both oral and parenteral considerations may apply in pharmaceutical contexts. Accordingly, absorption, distribution, metabolism, and excretion (ADME) data, skin irritation and sensitization studies, and formulation-specific stability assessments are incorporated as required. From a systems-level perspective, scientific evaluation integrates compositional profiling, toxicological data, exposure modelling, and intended-use classification to ensure that biological variability, processing conditions, and formulation context are coherently reflected in regulatory decision-making. This flexible yet structured evaluation framework enables consistent scientific assessment while accommodating sector-specific regulatory expectations, reinforcing the safe and responsible industrial translation of microalgal HVPs.

#### 4.1.3 Exposure assessment and use-level considerations

Exposure assessment is a central component of microalgal HVP safety evaluation and is inherently application-specific, reflecting differences in intake patterns, formulation, and routes of exposure across food, cosmetic, and pharmaceutical sectors. It provides the quantitative link between hazard identification and risk characterisation by integrating concentration data with use patterns and population-specific consumption scenarios. For food and nutraceutical uses, historical consumption provides an important comparative benchmark. Species such as *Spirulina* and *Chlorella* have long-standing dietary use, supporting typical intake ranges of approximately 1–10 g/day in humans without significant adverse effects (Verma et al., 2024; Wang C.-A. et al., 2024). This history of safe consumption informs margin-of-exposure calculations and supports risk-proportionate regulatory evaluation. This history of use has facilitated regulatory acceptance and streamlined safety assessments for these products. In contrast, HVPs derived from species such as *Nannochloropsis* or *H. pluvialis*, particularly concentrated carotenoids and omega-3 lipids, lack extensive traditional consumption records. Their evaluation therefore relies on controlled toxicological studies, clinical data, and exposure modeling conducted under novel food or ingredient frameworks (EU2, 2015). Exposure modeling typically incorporates estimated daily intake (EDI), body-weight–adjusted dose calculations, and uncertainty factors to establish acceptable intake levels. These data support defined intake limits for compounds such as beta-carotene, astaxanthin, EPA, and DHA, which are generally well below thresholds associated with adverse effects.

Beyond oral exposure, cosmetic and pharmaceutical applications require additional consideration of dermal or targeted therapeutic exposure. For topical products, safety assessments emphasize skin irritation, sensitization, dermal absorption potential, and cumulative exposure from repeated application, while pharmaceutical uses may involve higher, short-term doses subject to stricter clinical oversight. In pharmaceutical contexts, exposure assessment may extend to pharmacokinetic profiling, therapeutic index evaluation, and benefit–risk analysis under controlled clinical conditions. Across all sectors, combined exposure from multiple dietary or consumer sources is evaluated to ensure cumulative intake remains within established safety margins. This cumulative exposure assessment is particularly relevant for widely marketed omega-3 supplements and carotenoid-fortified products, where concurrent consumption may occur.

Population-specific considerations, including pregnancy, metabolic disorders, allergies, or concurrent supplementation, necessitate tailored risk communication, labeling, and use recommendations (Cruz and Vasconcelos, 2024). Vulnerable subpopulations may require additional safety factors or restricted use-level recommendations depending on the compound and exposure route. Overall, available evidence indicates that, when produced under controlled conditions and used within defined limits, microalgal HVPs exhibit favorable exposure profiles across food, cosmetic, and pharmaceutical applications. From a systems-level perspective, early integration of exposure assessment into strain selection, formulation design, and regulatory strategy enhances industrial predictability and reduces late-stage compliance barriers. This flexibility underscores their suitability for diverse bioindustrial markets, provided exposure assessment is integrated early into product development and regulatory strategy.

### 4.2 Regulatory frameworks and safety assessment of microalgal HVPs

Regulatory oversight of HVPs differs across jurisdictions but converges on shared core principles, including strain-specific identification, controlled production processes, compositional consistency, and evidence-based safety evaluation. These principles reflect a risk-based regulatory philosophy in which hazard characterization, exposure assessment, and intended use collectively determine approval pathways. In the European Union, Regulation (EU) 2015/2283 governs food and feed applications, requiring comprehensive dossiers covering taxonomic identity, manufacturing conditions, toxicological data, and exposure assessment. Dossiers typically include detailed specifications, stability data, contaminant analysis, and validated analytical methodologies to ensure batch reproducibility. Cosmetic and pharmaceutical applications are regulated under sector-specific legislation with additional emphasis on dermal exposure, formulation stability, and clinical relevance. In Saudi Arabia, the SFDA applies comparable requirements across food, cosmetic, and pharmaceutical categories, prioritizing microbial safety, contaminant limits, and validated process controls. This cross-sector regulatory alignment underscores the importance of integrating safety validation early in process development.

In the United States, regulatory evaluation is coordinated primarily by the FDA through GRAS notifications for food ingredients, safety substantiation for cosmetics, and established drug approval pathways for pharmaceutical applications. GRAS determinations require publicly available safety evidence and expert consensus, whereas pharmaceutical approvals demand comprehensive preclinical and clinical data under Good Manufacturing Practice (GMP) compliance. Despite differences in regulatory structure and documentation, EFSA, SFDA, and FDA all emphasize traceable strain identity, reproducible cultivation conditions, and robust safety data as prerequisites for market authorization.

A key regulatory challenge arises from the strain-resolved nature of microalgal approvals. Safety authorization is typically restricted to defined strains, cultivation modes, and processing conditions, limiting data transferability across producers. Consequently, even closely related strains may require independent safety evaluation if genetic background, cultivation inputs, or processing parameters differ. While this approach strengthens consumer protection and product consistency, it increases regulatory burden for emerging HVPs. Standardization of analytical methods, harmonized safety benchmarks, and structured data-sharing frameworks could facilitate partial mutual recognition while preserving risk-based oversight. Harmonization of analytical methods and partial mutual recognition frameworks could accelerate market entry without compromising safety.

From an industrial biotechnology perspective, regulatory preparedness and safety assurance are determinants of commercial viability that are as critical as biological productivity and process efficiency. Regulatory readiness influences investment decisions, market access timelines, and long-term scalability. Microalgal HVPs benefit from high traceability and reduced contamination risk, particularly when produced in closed or semi-closed systems. However, pronounced strain-specific diversity necessitates product- and strain-resolved safety frameworks. Alignment of strain engineering, cultivation optimization, quality control systems, and regulatory strategy therefore constitutes a core component of industrial design rather than a downstream administrative requirement. When rigorous regulatory alignment progresses in parallel with advances in mixotrophic cultivation, strain engineering, and downstream processing, microalgae can function as safe, scalable, and regulation-ready biofactories across food, cosmetic, and pharmaceutical value chains.

## 5 Discussion and challenges

Microalgae present a strong platform for producing HVPs, yet their large-scale industrial deployment remains constrained by interconnected biological, technical, economic, and regulatory challenges. These constraints interact across the value chain, amplifying scale-up complexity and investment risk rather than operating in isolation. Although earlier sections highlight significant advances in cultivation strategies, strain engineering, and downstream processing, persistent bottlenecks continue to limit commercial translation. A primary limitation is the gap between theoretical and achieved productivity. Laboratory-scale yields obtained under tightly controlled conditions frequently decline under industrial irradiation gradients, hydrodynamic stress, and nutrient variability. Conventional photoautotrophic systems suffer from light inefficiencies and environmental variability, while closed photobioreactors improve control at the expense of higher capital and energy inputs. This trade-off between biological precision and techno-economic feasibility remains a central design dilemma in microalgal bioprocess development. Mixotrophic cultivation offers a productive alternative by combining photosynthetic and heterotrophic metabolism, but requires careful control of carbon inputs to avoid growth inhibition and metabolic imbalance. Predictive modelling and real-time metabolic monitoring are therefore increasingly necessary to stabilize productivity under industrial conditions. Genetic and metabolic engineering have enhanced photosynthetic efficiency, stress tolerance, and carbon flux toward target metabolites in species such as *Phaeodactylum tricornutum* and *H. pluvialis*; however, these biological gains translate into industrial value only when supported by scalable cultivation, robust harvesting, and reliable extraction systems.

Safety and contamination risks represent an additional barrier, driven by the strain-specific biochemical diversity of microalgae. While many production strains are safe and well-characterized, closely related taxa may produce cyanotoxins or accumulate heavy metals, particularly in open systems. Metabolic plasticity under stress conditions may also alter secondary metabolite profiles, necessitating continuous compositional monitoring. Molecular strain identification, closed cultivation platforms, and targeted strain engineering have improved safety control, but broader adoption is often limited by cost, regulatory scrutiny, and operational complexity.

Economic and scalability constraints further shape feasibility. Advanced cultivation systems, green extraction technologies, and comprehensive analytical testing substantially increase production costs. Capital expenditure (CAPEX), operational expenditure (OPEX), and energy intensity collectively determine whether premium market positioning can offset production costs. Although strategies such as cell wall modification, waste-derived nutrient utilization, process intensification, and hybrid reactor designs show promise for cost reduction, their industrial performance requires validation under long-term continuous operation. Techno-economic modelling and life cycle assessment (LCA) are increasingly essential to substantiate sustainability claims and guide strategic investment decisions. In parallel, regulatory fragmentation remains a critical challenge. Strain-specific approval requirements under frameworks such as the EU and SFDA novel products regulation increase development timelines, particularly for genetically engineered or novel strains lacking historical consumption data. Regulatory preparedness must therefore be incorporated into early-stage strain selection and process design to prevent costly late-stage reformulation or revalidation.

Overall, these challenges reinforce a central conclusion of this review: successful commercialisation of microalgal HVPs depends on strategic alignment across strain design, cultivation strategy, downstream processing, safety validation, techno-economic assessment, and regulatory governance. Incremental refinement of isolated process components is unlikely to deliver sustained industrial competitiveness. Progress toward cohesive biorefinery models, supported by predictive modelling, data-informed metabolic engineering, and harmonised regulatory pathways, will be essential to position microalgae as reliable, scalable, and regulation-ready biofactories within the global bioeconomy.

## 6 Conclusion and future outlook

Microalgae have progressed from exploratory biomass resources to increasingly viable industrial platforms for the production of HVPs, including carotenoids, PUFAs, polysaccharides, and phycobiliproteins. This evolution reflects a transition from resource-driven biomass valorization toward precision, metabolite-focused biomanufacturing. This review adopted a product-oriented industrial biotechnology perspective to evaluate how biological performance, cultivation strategies, downstream processing, safety assessment, and regulatory readiness collectively determine the feasibility of microalgal HVP commercialisation. By linking metabolic regulation with engineering constraints and regulatory determinants, the analysis advances beyond descriptive synthesis toward structured industrial assessment. By moving beyond biomass-centric analyses, it reflects the practical requirements of scalable deployment.

Among the major product classes, carotenoids and omega-3 PUFAs currently represent the most industrially mature microalgal HVPs. Their advancement is supported by established cultivation systems, regulatory approvals, and ongoing improvements in green and hybrid extraction technologies. These product classes demonstrate comparatively consolidated value chains in which strain optimization, controlled cultivation, extraction selectivity, stability management, and regulatory validation are functionally aligned. In contrast, polysaccharides and phycobiliproteins remain at an earlier stage of industrial readiness despite strong bioactivity and broad functional potential. Their advancement is constrained by metabolic variability, purification complexity, stability challenges, and less harmonized regulatory familiarity. The comparison illustrates that industrial maturity depends not solely on biological potential, but on alignment across strain selection, process design, downstream compatibility, techno-economic performance, and regulatory acceptance.

Innovative strategies such as controlled stress induction, mixotrophic cultivation, and genetic or gene-editing approaches have demonstrated clear potential to enhance HVP productivity and process robustness. However, biological optimization alone does not ensure industrial competitiveness. Upstream performance gains translate into commercial value only when supported by scalable, cost-effective, and regulation-compliant downstream operations. Extraction efficiency, product stability, solvent management, energy intensity, and quality assurance ultimately determine market viability. Downstream processing remains a dominant cost and sustainability bottleneck, underscoring the need for biorefinery concepts that align cultivation, extraction, purification, and formulation within a coherent design architecture. Isolated technological improvements are insufficient; sustained competitiveness requires coordinated optimization across metabolic regulation, reactor configuration, harvesting strategy, and compliance pathways.

Safety assessment and regulatory alignment have emerged as primary determinants of commercial success rather than secondary considerations. The strain-specific diversity of microalgae necessitates product- and strain-resolved safety frameworks that incorporate accurate identification, compositional and toxicological characterisation, exposure assessment, and regulatory compliance. Regulatory preparedness directly influences development timelines, capital allocation, and market access, positioning compliance strategy as a structural component of industrial planning. Persistent gaps in standardized analytical methods and regulatory harmonization continue to delay market entry for several promising HVPs, particularly emerging products and engineered strains. Early incorporation of safety validation, documentation readiness, and harmonized testing protocols during strain development can substantially reduce translational risk.

Looking ahead, progress in microalgal HVPs will depend on transitioning from descriptive biological exploration to structured bioindustrial manufacturing frameworks. Future competitiveness will rely on predictive metabolic modelling, multi-omics integration, dynamic cultivation control, and data-informed techno-economic evaluation. Priority directions include coupling mixotrophic cultivation with targeted strain engineering, advancing scalable and low-impact downstream technologies, and fostering harmonised regulatory pathways that support innovation while maintaining safety.

In summary, microalgal HVPs occupy a strategic position at the intersection of industrial biotechnology, sustainable bioprocessing, and regulatory science. Long-term success depends on coordinated value-chain optimization rather than isolated technological breakthroughs. Carotenoids and PUFAs provide established industrial benchmarks, while polysaccharides and phycobiliproteins represent the next frontier for product diversification. By synthesizing biological, technological, and regulatory dimensions within a structured industrial perspective, this review positions microalgae not merely as alternative biomass sources but as precision biofactories for high-value compounds, offering a consolidated reference to support scalable, compliant, and economically sustainable deployment.
